# Multi-parameter MRI-based model for the prediction of early recurrence of hepatitis B-associated hepatocellular carcinoma after microwave ablation

**DOI:** 10.3389/fcimb.2025.1638779

**Published:** 2025-08-27

**Authors:** Ying Zhang, Jing-Jing Yu, Wei Chen, Bo Liu, Xue-Fei Wei, Zhao-Hui Wang, Xue Li, Shuai Gao, Kai Wang

**Affiliations:** ^1^ Department of Hepatology, Qilu Hospital of Shandong University, Jinan, China; ^2^ School of Radiology, Shandong First Medical University and Shandong Academy of Medical Sciences, Jinan, China; ^3^ Department of Radiology, Qilu Hospital of Shandong University, Jinan, China

**Keywords:** radiomics, deep learning, MRI, HBV-HCC, microwave ablation

## Abstract

**Objectives:**

To establish and validate a multi-parameter model for the prediction of early recurrence in patients with hepatitis B-associated hepatocellular carcinoma (HBV-HCC) after microwave ablation.

**Methods:**

This study retrospectively reviewed the clinical features and preoperative magnetic resonance imaging (MRI) scans of 166 patients with HBV-HCC who underwent microwave ablation at two hospitals. The training cohort comprised 116 patients from the first hospital (n = 116; mean age, 56 years; 84 male patients), while 50 patients from the second hospital constituted the external validation cohort (n = 50; mean age, 60 years; 38 male patients). A transformer-based deep learning network was used to fuse images from multi-sequence MRI and predict recurrence within 1 year after microwave ablation. Additionally, a nomogram based on deep learning radiomics and clinical features was developed and externally validated in a validation group from a second hospital.

**Results:**

The combined model was better than the clinical model and MRI model in predicting early recurrence of hepatitis B-associated hepatocellular carcinoma within 1 year after microwave ablation. Nomograms based on joint models include aspartate aminotransferase, portal hypertension, and deep learning-based radiomics scores. The areas under curves of the models in the training group and the validation group were 0.868 (95% CI: 0.793–0.924) and 0.842 (95% CI: 0.711–0.930), respectively, indicating high prediction ability. The results of decision curve analysis showed that the combined model had good clinical application value and correction effect.

**Conclusions:**

Our nomogram combined with clinical features and preoperative magnetic resonance imaging features effectively predicted early recurrence of hepatitis B-associated hepatocellular carcinoma within 1 year after microwave ablation.

## 1 Introduction

Primary liver cancer is one of the most common tumors worldwide, ranking sixth in incidence and third in terms of cancer-related mortality (Bray et al., 2024). Hepatocellular carcinoma (HCC) is the main type of primary liver cancer (75.0–85.0%), and its main cause is hepatitis B virus (HBV) infection (de Martel et al., 2020). HBV infection can lead to chronic inflammation of the liver and cirrhosis. The risk of developing HCC for patients with chronic HBV infection is 5 to 15 times higher than that of the general population; moreover, 70.0–90.0% of patients with cirrhosis after HBV infection will develop HCC (El-Serag and Rudolph, 2007). In patients with early HCC, surgical resection, liver transplantation, and local ablation are available treatment modalities. Local ablation mainly includes microwave ablation and radiofrequency ablation. Studies have shown that microwave ablation achieves overall survival and disease-free survival rates that are comparable to those of surgical treatment. However, it offers additional advantages, including fewer complications and lower hospitalization costs. Additionally, patients undergoing microwave ablation typically have shorter hospital stays (Wang et al., 2022). Although microwave ablation is a very effective treatment modality for HCC, the risk of early recurrence after ablation is still a concern. Studies have shown that up to 70.0% of patients with HCC who have cirrhosis relapse within 5 years after surgical resection or microwave ablation, with the highest recurrence rate being within 2 years after ablation (Rimola et al., 2020).

Establishing an effective model to monitor early HCC recurrence after microwave ablation will facilitate the timely prediction of recurrence. This will allow clinicians to formulate treatment plans in advance and attenuate HCC progression. Such a model will also provide an accurate and reliable basis for treatment selection. Ultimately, it may improve HCC cure rates, reduce recurrence, and improve patients’ outcomes. Currently, there are several models for predicting the prognosis of HCC after microwave ablation; however, these models do not distinguish the etiology of HCC (Zhou et al., 2021; Wu JP. et al., 2022; Zhang et al., 2022; Wang et al., 2024). HBV infection is an important factor that affects the recurrence of HCC, and the prognosis of HCC varies based on the different etiologies (Chen et al., 2006; Wong et al., 2017; Wang et al., 2023). Therefore, the influence of different etiologies on HCC recurrence should be considered in the establishment of a prediction model.

In recent years, artificial intelligence has become an important strategic tool for the development of science and technology and has facilitated numerous breakthroughs in the medical field. It can be used to extract information reflecting functional classification and prognostic value from medical data, making it possible to predict the intelligent risk of diseases and determine disease prognosis. Radiomics enables the extraction of large-scale quantitative image features, performing automated tumor segmentation and predictive model development through advanced data mining techniques. By deeply analyzing these extracted image characteristics, the technique provides valuable decision-support tools for clinical practice. This approach can assist physicians in achieving more accurate diagnostic assessments and planning treatments to improve patients’ outcomes (Xia et al., 2024). Current prognostic models for HCC after surgery are predominantly Computed Tomography (CT)-based (Ji et al., 2019; Ji et al., 2020; Yan et al., 2024), while Magnetic Resonance Imaging (MRI)-based studies remain limited and are mostly single-center studies (Gao et al., 2022; Yu Y. et al., 2022; Kuang et al., 2024). These factors have led to constrained generalizability and external validity of existing findings. Therefore, this study aimed to establish an early recurrence prediction model of HBV-HCC after microwave ablation based on a deep learning method to predict early recurrence in patients and provide a reference for the formulation of clinical protocols.

## 2 Methods

### 2.1 Workflow

**Figure 1:**
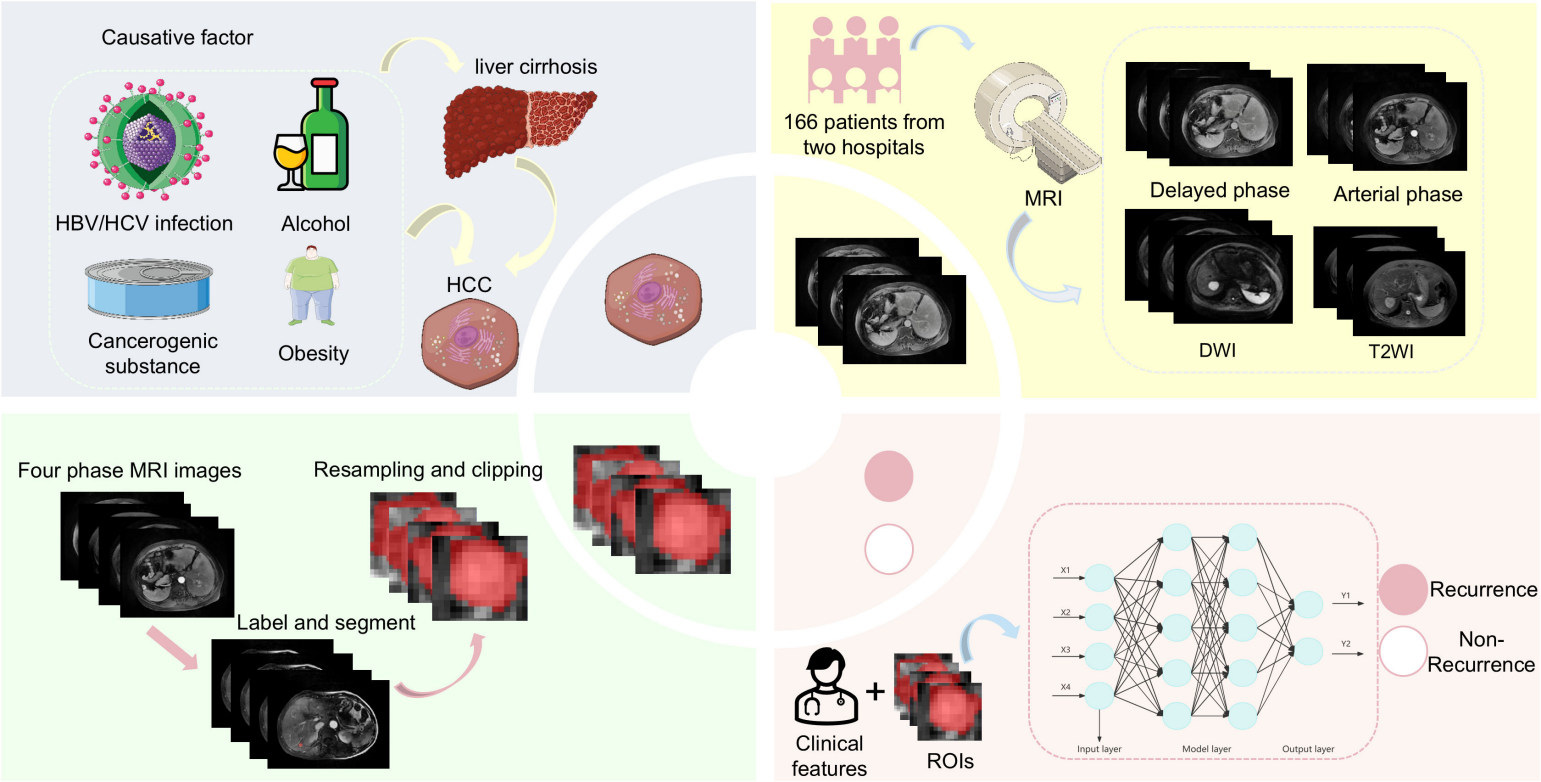
Study flowchart.

**Figure 2:**
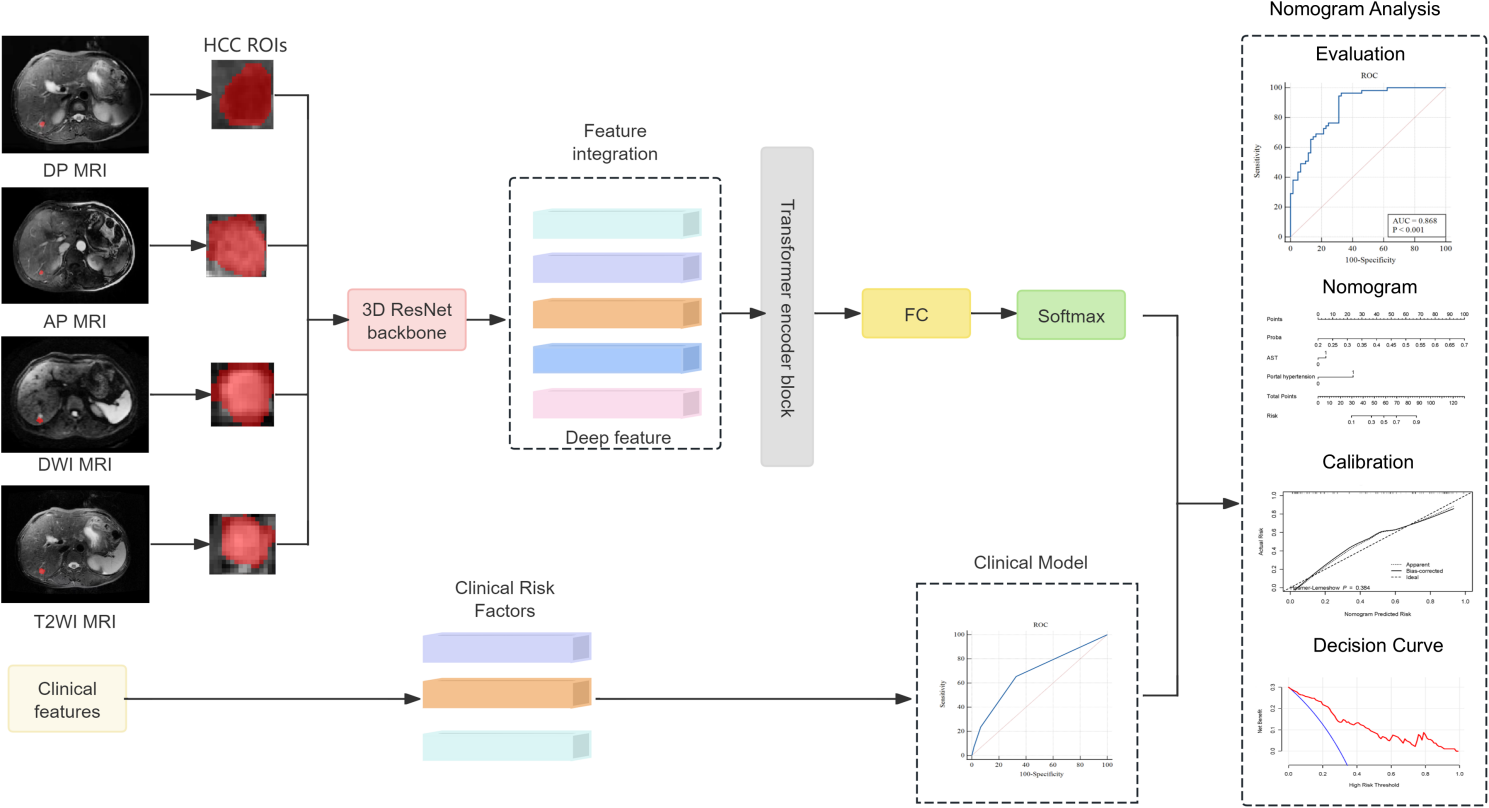
The process of establishing the Nomogram analysis in HBV-HCC.

The research process of this study is shown in **Figures 1**, **2**. The research process comprised three steps: (1) image segmentation; (2) image feature extraction and model construction; and (3) nomogram model construction using clinical features and imaging omics.

### 2.2 Patients

**Figure 3:**
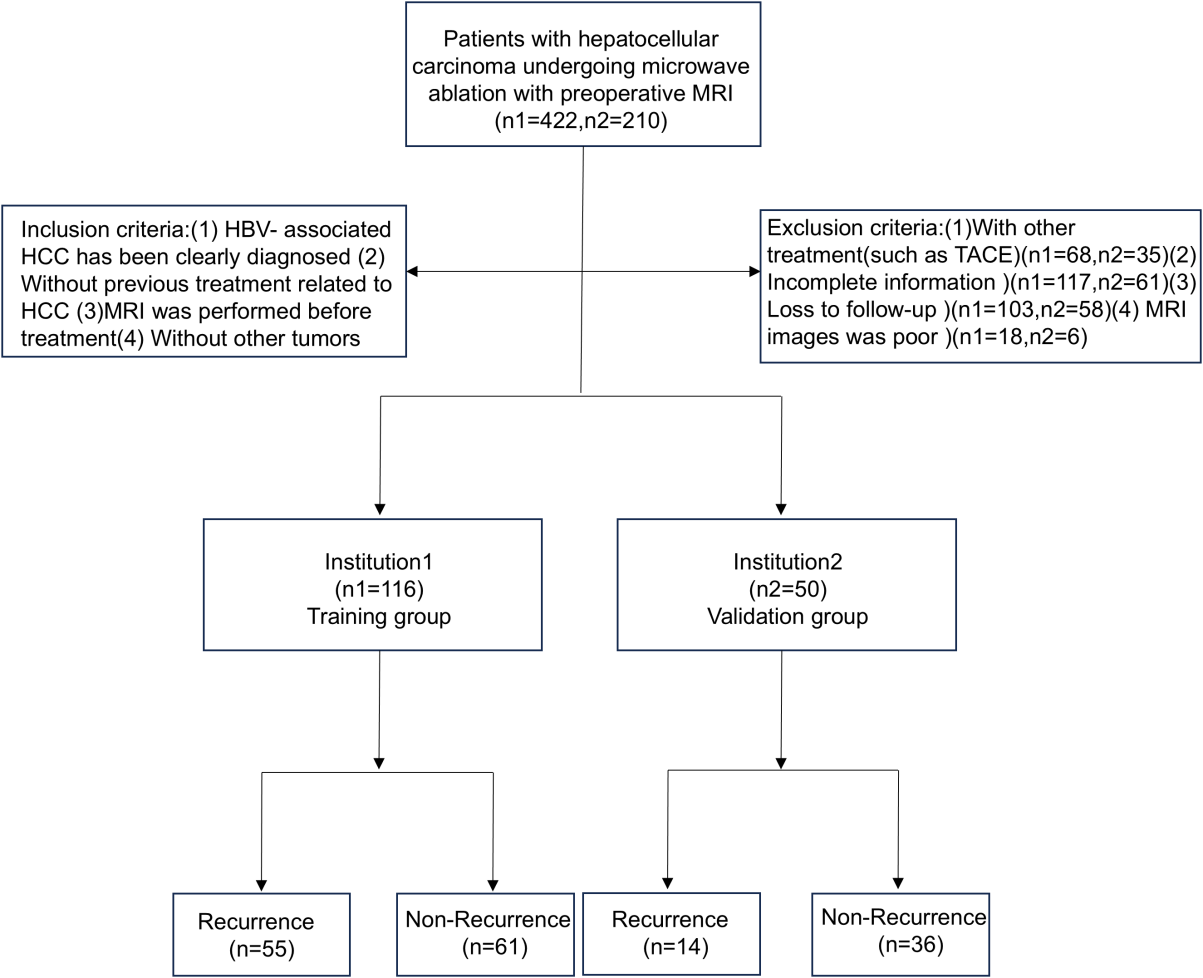
Flow chart of patients’ enrollment.

The study included patients from Qilu Hospital of Shandong University and Shandong Provincial Hospital who were hospitalized between January 1, 2017, and December 30, 2020. All patients were diagnosed with HBV-HCC and underwent percutaneous microwave ablation. The inclusion criteria are as follows: (1) patients in whom HBV-HCC was clearly diagnosed; (2) without previous treatment related to HC; (3) MRI was performed before treatment; and (4) absence of other tumors. HBV-HCC was diagnosed according to the 2018 Practice Guidance by the American Association for the Study of Liver Diseases (AASLD) (Marrero et al., 2018). The exclusion criteria are as follows: (1) patients who had received other treatments, such as transcatheter arterial chemoembolization (TACE); (2) patients with incomplete information; (3) those that were lost to follow-up; and (4) cases with poor MRI images. The specific process is shown in **Figure 3**.

The primary endpoint was early recurrence (ER), defined as the time from the start of treatment to recurrence within 1 year. This retrospective study was approved by the Ethics Committee of Shandong University Qilu Hospital (No. KYLL-2022(ZM)-961) and Shandong Provincial Hospital (SWYX: No. 2022-079), and the requirement for written informed consent was waived.

### 2.3 MRI acquisition and region of interest segmentation

All patients were scanned using MRI (1.5T, MAGNETOM Aera, Siemens Healthcare, Erlangen, Germany) before surgery using a 16-channel abdominal coil to receive signals. The patient was placed in the supine position. The head was inserted into the machine first, and the examination scope covered the entire liver. A T2WI coronal scan was performed, followed by an axial breath-hold fat suppression sequence T2WI (scan parameters: repetition time (TR), 3000–4000 ms; echo time (TE), 90–104 ms; slice thickness, 5 mm; slice interval, 0.5 mm). Dynamic contrast-enhanced scanning used axial breath-hold volume interpolated breath-hold examination (VIBE) (TR: 3.92 ms, TE: 1.39 ms, flip angle: 9°, slice thickness: 5 mm). The acquisition included T2-weighted imaging (T2WI), T1-weighted imaging (T1WI), and diffusion-weighted imaging (DWI). Images of the arterial phase (AP), portal venous phase (PVP), and delayed phase (DP) were obtained at 35 s, 50–65 s, and 5–8 min after the injection of the contrast agent. We collected the required scanning sequence AP, DP, T2WI, and DWI. Furthermore, AP, DP, T2WI, and DWI scan images of the patient’s MRI were obtained from the PACS system (DICOM format) and uploaded to ITK-SNAP software (Version 4.0.0-alpha-3). The radiologist delineated the region of interest (ROI) on the four-phase images along the lesion edges. The ROI mapping was performed independently by two radiologists with 6 years (physician 1) and 8 years (physician 2) of experience in abdominal imaging diagnosis, respectively. The radiologists were blinded to the purpose and content of the study. If the two physicians disagreed, they were asked to discuss the areas of disagreement and arrive at a consensus.

### 2.4 Deep learning-based radiomics score construction and validation

Before extracting the radiomics features, the AP, PVP, and DP images were preprocessed. Linear interpolation was used to resample the images to 1 mm × 1 mm × 1 mm to attempt to reduce the influence of different layer thicknesses. Afterwards, gray-level discretization processing was applied to convert the continuous images into discrete integer values to enhance the robustness of the imaging features (Wu C. et al., 2022). The inputs of the deep learning model had to be fixed-size images containing the corresponding tumor area; therefore, the MRI images were cropped using a 3D bounding box covering the tumor region. The tumor image was then scaled to a voxel size of 64 × 64 × 32 as the input of the model. For each MRI sequence, we built a 3D ResNet50 model to extract the deep learning-based radiomics (DLR) features. Afterwards, the DLR features were passed into a transformer network using multi-headed self-attention that considered each DLR feature as a token and related each element to every other element. Specifically, the image features (*f*DP, *f*AP, *f*DWI, and *f*T2WI) of the four sequences were concatenated to obtain Ftrans. After linear projections, the concatenated features are mapped to the key (K) vector, query (Q) vector, and value (V) vector, and a self-attention layer computed a query-key product as described below:


$$SA\left(Q,K,V\right)=softmax\left(\frac{QK^{T}}{{\sqrt{d}}_{k}}\right)V,$$

The correlation was calculated by the dot product between the Q vector and the K vector, and the attention score value was scaled to 0–1 using the Softmax operation and multiplied with V to obtain the optimized feature vector. A multi-head self-attention mechanism was then applied, and the outputs were fused in a weighted manner. After processing through the Transformer module, we obtain the image feature representation Ftrans, which contained *f*DP, *f*AP, *f*DWI, and *f*T2WI, and acted as the final feature of the image. Finally, we combined the feature connections and used a fully connected layer to compute the DLR score.

### 2.5 Development and validation of the combination nomogram

Logistic regression analysis was used to screen for potential clinical factors that may influence early recurrence after microwave ablation, such as sex, age, hepatitis B surface antigen (HBsAg), alpha-fetoprotein (AFP), alanine aminotransferase (ALT), aspartate aminotransferase (AST), gamma-glutamyl transferase (GGT), alkaline phosphatase (ALP), total bilirubin (TBIL), albumin (ALB), hypersplenotrophy, hypertension, tumor number, and tumor size. A combined model integrating the DLR score with independent clinical factors was subsequently established via multivariate logistic regression to enhance predictive accuracy. To facilitate clinical application, a nomogram was constructed based on the combined model, visualizing the contributions of each predictor through a graphical scoring system that translates individual patient data into a personalized probability of early recurrence within 1 year.

### 2.6 Statistical analysis

Clinical features were analyzed using the chi-square test or Fisher’s exact test, as appropriate. Univariate logistic regression analysis was performed to identify clinical factors associated with early recurrence after microwave ablation, with variables having p-values < 0.05 considered for inclusion in multivariate logistic regression to construct the clinical model. The deep learning-based radiomics features were extracted and fused as detailed in the methods section, culminating in the computation of the DLR score. Binary logistic regression was employed to develop the radiomics model, clinical model, and combined nomogram integrating the DLR score with selected clinical predictors. Model discriminatory performance was evaluated using the operating characteristic curve (ROC), calculating the area under the curve (AUC) with 95% confidence intervals (CI), along with sensitivity, specificity, accuracy, F1-score, positive predictive value (PPV), and negative predictive value (NPV). Model calibration was assessed using calibration curves. Clinical utility was determined using DCA to quantify net benefits across various threshold probabilities. All statistical analyses were performed using R software version 4.1.0 (R Foundation for Statistical Computing, Vienna, Austria), SPSS version 26.0 (IBM Corp., Armonk, NY, USA), and MedCalc version 20.010 (MedCalc Software Ltd., Ostend, Belgium).

## 3 Results

### 3.1 Clinical characteristics of patients

A total of 166 individuals were included in this study, including 116 in the training group and 50 in the validation group. There were 70 patients (42.2%) with HBV-HCC recurrence within 1 year after microwave ablation. A total of 116 patients were included in the training group, 55 (47.4%) of whom had recurrence within 1 year, while 15 (30.0%) of the 50 patients included in the validation group also had recurrence within 1 year. The clinicopathological features of the training group and the validation group are shown in **Table 1**; the differences between the two groups were not statistically significant (P < 0.05).

**Table 1:** Patients’ clinical features.

Characteristics (%)	Training group (n = 116)		*P*	Validation group (n=50)		*P*
	Recurrence (n = 55)	Non-recurrence (n = 61)		Recurrence (n = 15)	Non-Recurrence (n = 35)	
Sex			0.366			0.773
Male	42 (76.4)	42 (68.9)		11 (73.3)	27 (77.1)	
Female	13 (23.6)	19 (31.1)		4 (26.7)	8 (22.9)	
Age(years)			0.527			0.447
≥50	45 (81,8)	47 (77.0)		14 (93.3)	30 (85.7)	
<50	10 (18.2)	14 (23.0)		1 (6.7)	5 (14.3)	
HBsAg (IU/mL)			0.623			0.440
≥1000	47 (85.5)	54 (88.5)		13 (86.7)	27 (77.1)	
<1000	8 (14.5)	7 (11.5)		2 (13.3)	8 (22.9)	
AFP (ng/mL)			0.284			0.416
≥20	28 (50.9)	25 (41.0)		10 (66.7)	19 (54.3)	
<20	27 (49.1)	36 (59.9)		5 (33.3)	16 (45.7)	
ALT (U/L)			0.155			0.037*
≥50	15 (27.3)	10 (16.4)		4 (26.7)	2 (5.7)	
<50	40 (72.7)	51 (83.6)		11 (73.3)	33 (94.3)	
AST (U/L)			0.019*			0.021*
≥40	27 (49.1)	17 (27.9)		9 (60.0)	9 (25.7)	
<40	28 (50.9)	44 (72.1)		5 (40.0)	26 (74.3)	
GGT (U/L)			0.332			0.440
≥60	18 (32.7)	15 (24.6)		4 (26.7)	6 (17.1)	
<60	37 (67.3)	46 (75.4)		11 (73.3)	29 (82.9)	
ALP (U/L)			0.374			0.736
≥125	9 (16.4)	14 (23.0)		2 (13.3)	6 (17.1)	
<125	46 (83.6)	47 (77.0)		13 (86.7)	29 (82.9)	
TBIL (μmol/L)			0.386			0.083
≥21	14 (25.5)	20 (32.8)		6 (40.0)	6 (17.1)	
<21	41 (74.5)	41 (67.4)		9 (60.0)	29 (82.9)	
ALB (g/L)			0.557			0.174
≤40	17 (30.9)	22 (36.1)		5 (33.3)	19 (54.2)	
>40	38 (69.1)	39 (63.9)		10 (66.7)	16 (45.7)	
Hypersplenotrophy			0.610			0.804
Positive	35 (63.6)	36 (59.0)		8 (53.3)	20 (57.1)	
Negative	20 (36.4)	25 (41.0)		7 (46.7)	15 (42.9)	
Portal Hypertension			0.009*			<0.001*
Positive	13 (23.6)	4 (6.6)		9 (60.0)	4 (11.4)	
Negative	42 (76.4)	57 (93.4)		6 (40.0)	31 (88.6)	
Tumor number			0.233			0.736
>1	40 (72.7)	50 (82.0)		11 (73.3)	24 (68.6)	
≤1	15 (27.3)	11 (18.0)		4 (26.7)	11 (31.4)	
Tumor size (cm)			0.747			0.895
≥2	42 (76.4)	45 (73.8)		10 (66.7)	24 (68.6)	
<2	13 (23.6)	16 (26.2)		5 (33.3)	11 (31.4)	

HBsAg, hepatitis B surface antigen; AFP, alpha-Fetoprotein; ALT, alanine aminotransferase; AST, aspartate aminotransferase; GGT, gamma-glutamyl transferase; ALP, alkaline phosphatase; TBIL, total Bilirubin; ALB, albumin.*P<0.050.

### 3.2 Modeling of deep learning-based radiomics features

**Figure 4:**
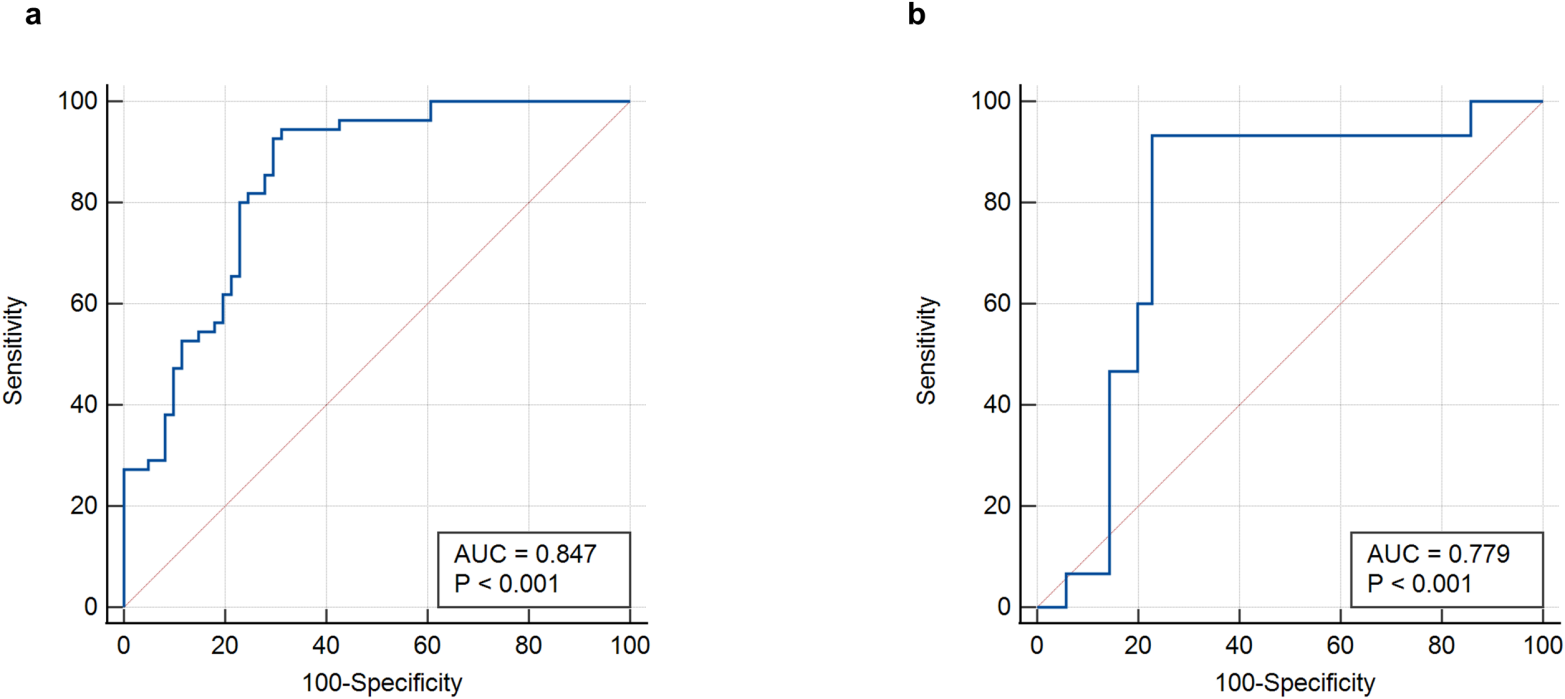
ROC curves of the DLR-score. **(a)** DLR-score in the training group. **(b)** DLR-score in the validation group. DLR, deep learning-based radiomics.

The ROC curve was used to evaluate the efficacy of the imaging model in predicting HBV-HCC recurrence within 1 year after microwave ablation. The DLR model we established in the training group had an AUC of 0.847 (95% CI: 0.768–0.907), a sensitivity of 94.5% (95% CI: 0.851–0.981), a specificity of 68.8% (95% CI: 0.564–0.791), an accuracy of 81.0%, an F1-score of 82.5%, a positive predictive value (PPV) of 73.3%, and a negative predictive value (NPV) of 93.3% in the training group (**Figure 4A**, **Table 2**). In the validation group, the AUC was 0.779 (95% CI: 0.639–0.884), sensitivity was 73.3% (95% CI: 0.480–0.891), specificity was 77.1% (95% CI: 0.610–0.879), accuracy was 76.0%, F1-Score was 64.7%, PPV was 57.9%, and NPV was 87.1% (**Figure 4B**, **Table 3**).

**Table 2:** Diagnostic value of different models in the training cohort.

	AUC (95% CI)	Accuracy (95% CI)	Sensitivity (95% CI)	Specificity (95% CI)	F1-Score (95% CI)	PPV (95% CI)	NPV (95% CI)
DLR model	0.847 (0.768–0.907)	0.810 (0.729–0.871)	0.945 (0.851–0.981)	0.688 (0.564–0.791)	0.825 (0.745–0.896)	0.732 (0.619–0.821)	0.933 (0.821–0.977)
Clinical model	0.681 (0.588–0.765)	0.663 (0.574–0.743)	0.655 (0.523–0.766)	0.672 (0.547–0.777)	0.649 (0.550–0.745)	0.643 (0.512–0.755)	0.683 (0.558–0.787)
Nomogram	0.868 (0.793–0.924)	0.810 (0.730–0.871)	0.963 (0.877–0.990)	0.672 (0.547–0.777)	0.828 (0.752–0.890)	0.726 (0.614–0.815)	0.953 (0.845–0.987)

DLR, deep learning-based radiomics; AUC, area Under curve; PPV, Positive Predictive Value; NPV, Negative Predictive Value.

**Table 3:** Diagnostic value of different models in the validation cohort.

	AUC (95% CI)	Accuracy (95% CI)	Sensitivity (95% CI)	Specificity (95% CI)	F1-Score (95% CI)	PPV (95% CI)	NPV (95% CI)
DLR model	0.779 (0.639–0.884)	0.760 (0.626–0.857)	0.733 (0.480–0.891)	0.771 (0.610–0.879)	0.647 (0.435–0.821)	0.579 (0.363–0.769)	0.871 (0.711–0.949)
Clinical model	0.771 (0.631–0.878)	0.800 (0.670–0.888)	0.600 (0.357–0.802)	0.886 (0.740–0.955)	0.642 (0.364–0.821)	0.692 (0.424–0.873)	0.837 (0.689–0.923)
Nomogram	0.842 (0.711–0.930)	0.820 (0.692–0.902)	0.800 (0.548–0.930)	0.838 (0.673–0.919)	0.727 (0.500–0.880)	0.667 (0.437–0.837)	0.901 (0.758–0.968)

DLR, deep learning-based radiomics; AUC, area Under curve; PPV, Positive Predictive Value; NPV, Negative Predictive Value.

### 3.3 Validation and evaluation of nomogram

**Figure 5:**
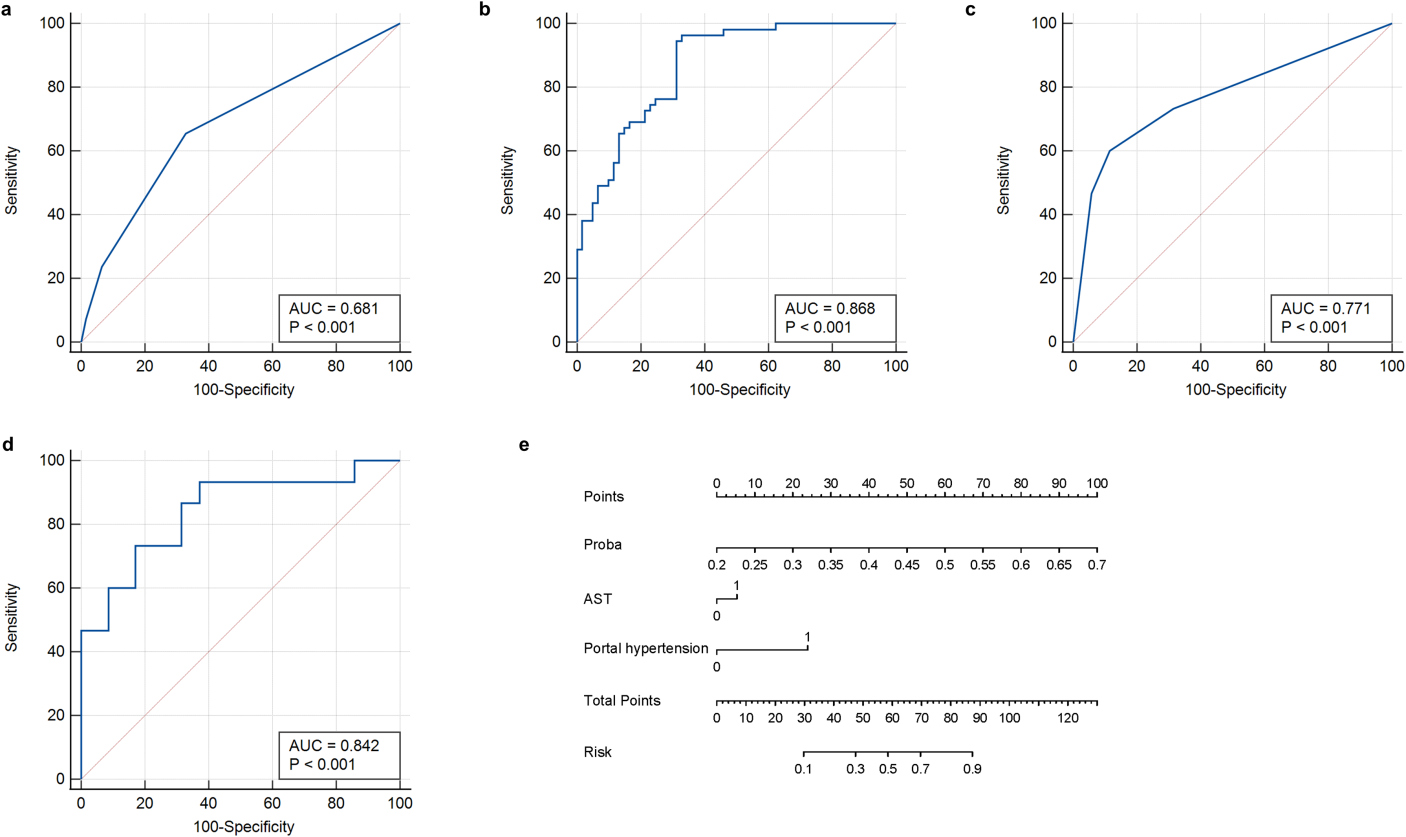
ROC curves and nomogram of the clinical-radiomics model. **(a)** Clinical feature model in training group. **(b)** Clinical-radiomics model in training group. **(c)** Clinical feature model in validation group. **(d)** Clinical-radiomics model in validation group. **(e)** Nomogram developed to predict recurrence. ROC, receiver operating characteristic curve.

Univariate logistic regression analysis was performed to identify clinical factors associated with early recurrence after microwave ablation; variables with p-values < 0.05, such as AST levels and portal hypertension, were considered for inclusion in the multivariate logistic regression to construct the clinical model (**Table 4**). A clinical prediction model was established with an AUC of 0.681 (95% CI: 0.588–0.765) and a sensitivity of 65.5% (95% CI: 0.523–0.766), a specificity of 67.2% (95% CI: 0.547–0.777), an accuracy of 66.3%, a F1-Score of 64.9%, a PPV of 64.3%, and an NPV of 68.3% (**Figure 5a**, **Table 2**). Similarly, in the validation group, the clinical model was established with an AUC of 0.771 (95% CI: 0.631–0.878), sensitivity of 60.0% (95% CI: 0.357–0.802), specificity of 88.6% (95% CI: 0.740–0.955), accuracy of 80.0%, F1-Score of 64.2%, PPV of 69.2%, and NPV of 83.7%. (**Figure 5c**, **Table 3**).

**Table 4:** Univariate and multivariate logistic regression of the clinical factors associated with early recurrence after HBV-HCC microwave ablation in training group.

Variables	Univariate analysis		Multivariate analysis	
	*p*	OR (95% CI)	*p*	OR (95% CI)
Male	0.367	1.462(0.641–3.335)		
Age (≥50 years)	0.527	1.340(0.540–3.326		
HBsAg (≥1000 IU/mL)	0.623	0.762(0.257–2.259)		
AFP (≥20 ng/mL)	0.285	1.493(0.716–3.113)		
ALT (≥50 U/L)	0.158	1.912(0.777–4.708)		
AST (≥40 U/L)	0.020	2.496(1.156–5.390)	0.008	5.299(1.555–18.055)
GGT (≥60 U/L)	0.333	1.492(0.663–3.355)		
ALP (≥125 U/L)	0.376	0.657(0.259–1.666)		
TBIL (≥17.1 μmol/L)	0.387	0.700(0.312–1.571)		
ALB (≤40 g/L)	0.557	0.793(0.365–1.721)		
Hypersplenotrophy	0.610	1.215(0.574–2.572)		
Portal Hypertension	0.014	4.411(1.343–14.490)	0.010	2.885(1.292–6.445)
Tumor number (>1)	0.236	1.705(0.705–4.118)		
Tumor size (≥2 cm)	0.747	0.871(0.374–2.025)		

OR, Odds Ratio.

Afterwards, we used the deep learning DLR score, AST, and portal hypertension as factors for the multivariate logistic regression analysis to build personalized prediction models. According to the results of the multivariate logistic analysis, two independent clinical factors were combined with DLR scores to establish a clinical-radiological model. The combined model showed the best evaluation efficacy in both cohorts, with AUC, sensitivity, and specificity in the training group being 0.868 (95% CI: 0.793–0.924), 96.3% (95% CI: 0.877–0.990), and 67.2% (95% CI: 0.547–0.777), respectively (**Figure 5b**, **Table 2**). The AUC, sensitivity, and specificity of the validation group were 0.842 (95% CI: 0.711–0.930), 80.0% (95% CI: 0.548–0.930), and 83.8% (95% CI: 0.673-0.919), respectively (**Figure 5d**, **Table 3**). In terms of accuracy, the nomogram also outperformed other models (training group, 81.0% vs. DLR: 81.0%, clinical: 66.3%; validation group, 82.0% vs. DLR: 76.0%, clinical: 80.0%). In addition, the F1-score was highest for the nomogram (training group, 0.828, vs. DLR: 0.825, clinical: 0.649; validation group, 0.727 vs. DLR: 0.647, clinical: 0.642), reflecting its balanced performance in imbalanced data scenarios. Regarding negative predictions, the nomogram also demonstrated the best NPV in both the training group (95.3% vs. DLR: 93.3%, clinical: 68.3%) and the validation group (90.1% vs. DLR: 87.1%, clinical: 83.7%), indicating high reliability in ruling out the target condition. The nomogram emerged as the most robust tool, combining high discriminative power (AUC) with balanced sensitivity-specificity profiles. Finally, we visualized the model as a nomogram for clinicians (**Figure 5e**).

### 3.4 Performance of the combination nomogram

**Figure 6:**
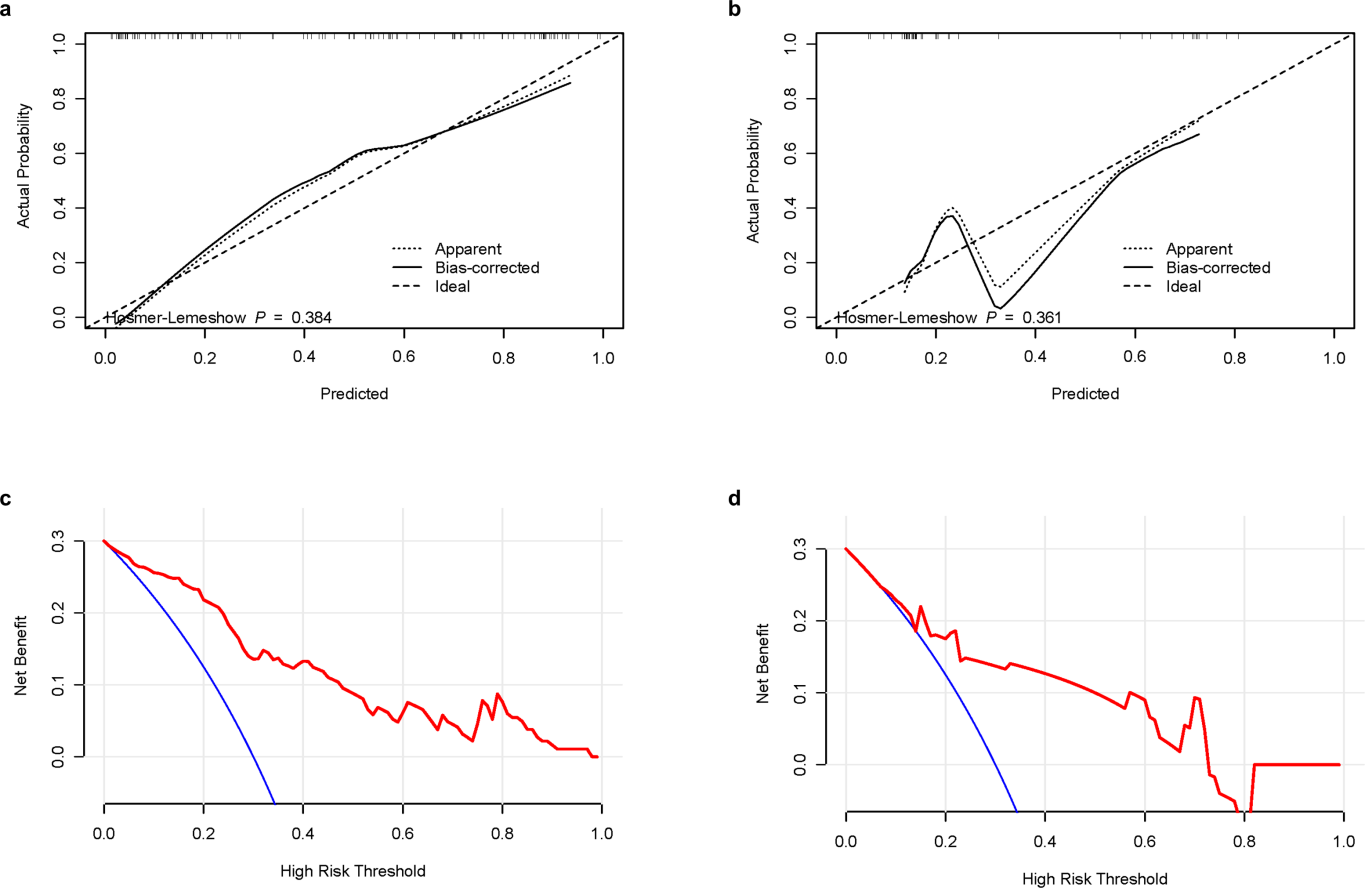
Calibration curves and DCA curves of the combination nomogram. **(a)** Calibration curves in the training group. **(b)** Calibration curves in the validation group. **(c)** DCA curves in the training group. **(d)** DCA curves in the validation group. DCA, decision curve analysis.

A calibration curve and the Hosmer-Lemeshow goodness-of-fit test showed that the predicted value and the actual result had a good fit in the training and validation groups (**Figures 6a, b**). We used decision curve analysis (DCA) to evaluate whether this nomogram could contribute to clinical treatment strategies. The DCA curves (**Figures 6c, d**) showed that the nomogram had a good net benefit in predicting HBV-HCC recurrence within 1 year after microwave ablation at most threshold ranges of 0.1–1.0. This indicates that patients with HBV-HCC can benefit immensely from the use of this nomogram.

## 4 Discussion

HCC is one of the leading causes of cancer-related death worldwide, with HBV infection being a major cause of HCC. In China, where hepatitis B prevalence is high, more than 80% of HCC cases are associated with HBV infection (WHO Guidelines Approved by the Guidelines Review Committee, 2015). Although HBV is currently being effectively controlled, completely curing HBV-HCC is still challenging (He et al., 2024). Although there have been great advances in the treatment of HCC, the prognosis of HCC remains poor due to its high recurrence rate and high metastasis rate. Currently, the recurrence rate after radical treatment was 50–80% (Ikai et al., 2007). Liver transplantation is the most effective method for early HCC; however, it is only beneficial for a small group of patients due to its high cost, strict indications, and limited access to liver sources (Zheng et al., 2020). In recent years, microwave ablation has received increasing attention from clinicians because of its minimal trauma, faster recovery, fewer complications, and shorter hospital stay (Yu J. et al., 2022). Microwave ablation also requires regular and effective follow-up monitoring, similar to surgical resection. However, it still carries a high recurrence risk, particularly within the first 2 years post-procedure (Rimola et al., 2020). Therefore, establishing a recurrence prediction model for patients with HBV-HCC after microwave ablation could enable early identification of high-risk cases. This would facilitate personalized treatment strategies and potentially reduce recurrence rates. In this study, deep learning methods were used to extract deep features from multimodal MRI before microwave ablation of early liver cancer; a transformer model based on cross-modal fusion was also constructed to calculate the DLR score. Combined with clinical indicators, a comprehensive nomogram was established to predict the recurrence of HBV-HCC after microwave ablation.

In recent years, with the development of artificial intelligence, several studies have explored the use of imaging omics to establish predictive models to predict HCC recurrence after microwave ablation. Wu et al. applied the convolutional neural network ResNet 18 and Pyradiomics to analyze gray ultrasound images, combined with clinical indicators, and established a prediction model to predict the prognosis and differentiation degree of HCC (Wu JP. et al., 2022). A 2022 study that developed a response algorithm based on multi-parameter MRI and clinical variables, retrospectively analyzed 339 patients to predict recurrence after microwave ablation of HCC (Zhang et al., 2022). Yuan et al. extracted a set of 647 radiomics features from enhanced computed tomography images and combined them with clinical features to build a model for predicting HCC recurrence after ablation (Yuan et al., 2019). However, these studies failed to differentiate HCC cases based on etiology, as they either relied solely on a single-center internal validation without external verification. Additionally, their use of convolutional neural network algorithms limited effective simulation of global and remote semantic interactions. However, we developed a transformer-based method for predicting HCC recurrence after microwave ablation, and our results indicate that it achieved a high predictive accuracy. In the training set, the AUC of the established imaging module DLR score was 0.847, and the AUC of the verification group was 0.779. Moreover, this study incorporated data from two research centers to enhance the model’s reliability. One center serves as the modeling group, while the other functions as the verification group, ensuring the stability, feasibility, and accuracy of the predictive model.

Furthermore, to achieve full fusion of MRI multi-modal information and avoid loss of tumor region details, we applied a deep learning transformer model. With its powerful global information capture capability and self-attention mechanism, the transformer has demonstrated outstanding performance in clinical image processing. By adjusting the network structure, enhancing the feature extraction ability, and optimizing the self-attention mechanism, the model improved the performance of information fusion, detail processing, and work efficiency (Han et al., 2023). We developed a deep learning-based prognostic model for early HCC recurrence post-microwave ablation that was capable of accurately predicting 1-year recurrence outcomes through comprehensive multi-modal image analysis. This model can assist clinicians in making more precise diagnostic and therapeutic decisions regarding microwave ablation treatment strategies.

Nomograms, a combination of omics and clinical markers, have been widely used to predict the prognosis of HCC. In this study, a prognostic model for HBV-HCC was also constructed based on deep learning and clinical factors. Consistent with the findings of previous studies, AST levels and portal hypertension were identified in this study as risk factors for postoperative recurrence of HCC (Xu et al., 2019; Liu et al., 2023). In this study, the AUC of the predictive model constructed by clinical factors in the modeling group and the validation group was 0.681 and 0.771, respectively, while the AUC was significantly improved when combined with the DLR-SCORE, as they increased to 0.868 and 0.842, respectively. This suggests that DLR-SCORE can improve the diagnostic performance of models constructed of clinical factors. Therefore, our proposed nomogram for predicting recurrence post-microwave ablation in patients with HBV-HCC could be a potential tool for preoperative evaluation.

The traditionally recognized risk factors for HCC recurrence, such as AFP level and tumor size, have indeed been widely validated in various clinical studies (He et al., 2018; He et al., 2023). However, in our cohort, these variables were not statistically significant in the multivariate analysis. We believe there might be the following reasons. First, there might be cohort characteristics and selection bias. Our study exclusively included HBV-HCC patients who underwent microwave ablation and had relatively early-stage tumors based on inclusion criteria. The range of tumor sizes was narrow, mostly ≤ 3 cm. Several patients had AFP levels that were within the normal limits or only slightly elevated, possibly due to early detection through regular surveillance in HBV-endemic regions. These restrictions reduced the variability and statistical power of AFP and tumor size in the multivariate model. Second, these variables might not have been significant in this study due to the unique biological effects of ablation. Microwave ablation induces tumor necrosis through thermal coagulation, and its effectiveness may be more dependent on technical factors, such as ablation margin and energy deposition, and the peritumoral microenvironment, rather than tumor size. In small HCCs (≤ 3 cm), complete ablation is often technically achievable, potentially blunting the predictive value of tumor size on early recurrence. Third, the outstanding predictive ability of radiomics features. Our transformer-based deep learning model extracted multi-parametric radiomic features from preoperative MR images, capturing both intra-tumoral heterogeneity and subtle peritumoral changes not reflected by tumor size alone. These high-dimensional features likely absorbed the variance traditionally explained by size and AFP, resulting in a lower relative contribution of these classic markers in the final model. Fourth, statistical considerations may have influenced our analysis. In multivariable analysis, collinearity and feature selection via regularization or stepwise methods may result in classic variables (e.g., AFP) being excluded in favor of stronger, non-redundant predictors such as radiomics scores. This does not imply that AFP or tumor size were irrelevant but rather that in this specific, early-stage, and treatment-homogeneous cohort, their incremental value was limited. Therefore, our results suggest that deep learning-based radiomics features, when integrated with key clinical parameters, such as liver function and portal hypertension, can outperform traditional single clinical indicators in predicting early recurrence. This highlights a potential change in basic assumptions towards imaging-based, data-driven risk stratification in the context of curative ablation.

### 4.1 Limitations

Our study has some limitations. First, this was a retrospective study. Prospective studies have better control of confounding factors and bias; therefore, we aim to design prospective studies in the future to further verify our conclusions. Second, while our external validation cohort (n=50) was well-characterized, its modest size and single-institution origin may limit its generalizability across diverse populations or imaging protocols. Multicenter validation is essential to confirm robustness, particularly for rare subtypes or borderline cases. Preliminary cross-institutional data (not included here) also suggest reproducible performance, supporting the scalability of our approach. To address this, we have initiated a prospective multicenter trial to pool data from heterogeneous sources, ensuring broader applicability. Beyond validation, seamless clinical integration is critical. We aim to develop an implementation framework to embed the nomogram as a DICOM-compatible tool within PACS, enabling automated risk score generation alongside radiology reports. Feedback from clinicians (e.g., via Likert-scale surveys) will guide usability refinements, ensuring practical utility. Regulatory considerations will also be addressed for clinical deployment. Third, in our study, the deep learning model was designed to process the ROI that encompasses the tumor, as annotated by the radiologists, allowing for image features to be learned effectively from the ROI. A key advantage of employing deep learning in radiomics analysis is its robustness to variations in boundary delineation. While inter-observer variability was not quantitatively assessed (e.g., via Dice similarity coefficient or Kappa statistics) due to the model’s resilience to such variations, future studies could incorporate such evaluations for enhanced reproducibility. Additionally, we deliberately excluded the portal venous phase (PVP) due to inconsistent image quality and a lack of standardization across retrospective datasets, thereby ensuring model reliability and generalizability. Despite this omission, our model achieved strong performance (AUCs 0.868–0.842) by effectively leveraging AP, DP, T2WI, and DWI sequences, demonstrating their sufficiency for recurrence prediction. While we acknowledge the clinical value of PVP, its exclusion was justified in this real-world study; however, in future prospective studies, we will assess its incremental benefit under standardized protocols. Finally, in China, most HCC cases were caused by HBV infection; therefore, the participants included in this study were patients with HBV-HCC. Since the prognosis of HCC is different due to different etiologies, the model needs to be further validated in patients with HCC due to other etiologies prior to its large-scale application.

## 5 Conclusion

In summary, we developed a transformer-based deep learning method to mine the correlation between different sequence MRI data to calculate the DLR score. Afterwards, we established a nomogram that combines MRI and clinical factors to predict the recurrence of early hepatocellular carcinoma after microwave ablation. This provides valuable information for personalized treatment and optimized management and an accurate and reliable basis for clinical decision-making.

## Data Availability

The original contributions presented in the study are included in the article/supplementary material. Further inquiries can be directed to the corresponding author.
